# Overall survival after definitive chemoradiotherapy for patients with esophageal cancer: a retrospective cohort study

**DOI:** 10.1093/dote/doae047

**Published:** 2024-06-04

**Authors:** Charlène J van der Zijden, Anna Bouwman, Bianca Mostert, Joost J M E Nuyttens, Pieter C van der Sluis, Manon C W Spaander, Jan Willem M Mens, Marjolein Y V Homs, Leni van Doorn, Bas P L Wijnhoven, Sjoerd M Lagarde

**Affiliations:** Department of Surgery, Erasmus MC Cancer Institute, Rotterdam, The Netherlands; Department of Surgery, Erasmus MC Cancer Institute, Rotterdam, The Netherlands; Department of Medical Oncology, Erasmus MC Cancer Institute, Rotterdam, The Netherlands; Department of Radiation Oncology, Erasmus MC Cancer Institute, Rotterdam, The Netherlands; Department of Surgery, Erasmus MC Cancer Institute, Rotterdam, The Netherlands; Department of Gastroenterology and Hepatology, Erasmus University Medical Center, Rotterdam, The Netherlands; Department of Radiation Oncology, Erasmus MC Cancer Institute, Rotterdam, The Netherlands; Department of Medical Oncology, Erasmus MC Cancer Institute, Rotterdam, The Netherlands; Department of Medical Oncology, Erasmus MC Cancer Institute, Rotterdam, The Netherlands; Department of Surgery, Erasmus MC Cancer Institute, Rotterdam, The Netherlands; Department of Surgery, Erasmus MC Cancer Institute, Rotterdam, The Netherlands

**Keywords:** definitive chemoradiotherapy, distant metastases, esophageal cancer, locoregional recurrence

## Abstract

Definitive chemoradiotherapy (dCRT) is a potentially curative therapy for esophageal cancer. As indications for dCRT differ widely, it is challenging to draw conclusions on outcomes and survival. The aim of this study was to evaluate overall survival (OS) and recurrence patterns according to indications for treatment. Patients who underwent dCRT (50.4 Gy concomitant with carboplatin/paclitaxel) for esophageal cancer between 2012 and 2022 were identified. Indications for dCRT were: cervical tumor, irresectable disease, unfit for surgery, and patient and/or physician preference. The primary endpoint was OS calculated with the Kaplan–Meier method. Secondary endpoints included the proportion of patients that completed the dCRT regimen, 30- and 90-day mortality, and disease recurrence. One hundred and fifty-seven patients were included (72.6% esophageal squamous cell carcinoma) with a median follow-up of 20 months (IQR 10.0–43.9). The full dCRT regimen was completed by 116 patients (73.9%). Thirty- and 90-day mortality were 2.5% and 8.3%, respectively. Median and 5-year OS for all patients were 22.9 months (95% CI 18.0–27.9) and 31.4%, respectively. The median OS per indication was 23.7 months (95% CI 6.5–40.8) for patients with cervical tumors, 10.9 months (95% 0.0–23.2) for irresectable disease, 28.2 months (95% CI 12.3–44.0) for unfit patients, and 22.9 months (95% CI 15.4–30.5) for patients’ preference for dCRT (*P* = 0.11). Disease recurrence was observed in 74 patients (46%), located locoregionally (46%), distant (19%), or combined (35%). Patients who underwent dCRT had a 5-year OS of 31.4%, but OS differed according to indications for treatment with patients who had irresectable disease having the worst prognosis.

## INTRODUCTION

Locally advanced esophageal cancer can be treated with neoadjuvant chemoradiotherapy (nCRT), followed by esophagectomy.^1^ Definitive chemoradiotherapy (dCRT) is an alternative treatment option that aims to eradicate the primary tumor and locoregional lymph nodes by using a higher total radiation dose (50.4 Gy).^2^^,^^3^ For cervical squamous cell carcinoma, dCRT is the preferred treatment, as surgery would include laryngopharyngectomy with a permanent tracheostomy. Other reasons for choosing dCRT are patients with an irresectable tumor (cT4b) and/or lymph node metastases, or patients with a poor performance status.^2–4^ Finally, there are patients who opt again surgery and prefer a non-surgical approach with curative intent.

Hence, the group of patients that undergo dCRT is heterogeneous, and a study from the Netherlands showed variations in the use of dCRT across hospitals.^5^^,^^6^

Previous studies reported locoregional (29%–45%) and distant recurrences (20%–41%) following dCRT.^7–9^ However, these studies did not give insight into the pattern of recurrence according to predefined indications for dCRT. It can be hypothesized that the pattern of disease recurrence differs according to indication. For example, more locoregional recurrences could be expected in patients with an irresectable tumor compared to patients undergoing dCRT for locoregional resectable disease.

Given the different indications and limited studies on dCRT, the aim of this study was to assess outcomes after dCRT according to the indication for dCRT in patients with esophageal cancer treated in a tertiary referral center.

## METHODS

### Study design and patients

This was a single-center retrospective cohort study involving all patients with esophageal cancer who were treated with curatively-intended dCRT at the Erasmus MC Cancer Institute between August 2012 and July 2022. Patients were identified from our institutional database. Inclusion criteria were: (i) histologically proven squamous cell carcinoma or adenocarcinoma of the esophagus or esophageal gastric junction (EGJ) and (ii) clinical staging with upper endoscopy with biopsies and CT-scan of the thorax and abdomen or^10^ F-FDG PET/CT. Patients with a concurrent diagnosis of other primary tumors were excluded. Since this was a retrospective study and most patients have died, the need for ethical approval and individual informed consent was waived by the Medical Ethical Committee of the Erasmus MC (MEC-2022-0348). The study was performed in accordance with the Declaration of Helsinki (64th World Medical Association General Assembly, Fortaleza, Brazil, October 2013).

### Definitive chemoradiotherapy

Treatment with dCRT consisted of 6 weekly cycles of intravenous paclitaxel (50 mg/m^2^) and carboplatin (AUC 2 mg/mL per min) with concurrent radiotherapy (50.4 Gy) in 28 fractions of 1.8 Gy on 5 days per week. No predefined follow-up protocol was implemented after completion of dCRT, and diagnostics were only performed in cases of complaints.

### Predefined indications

The indications for dCRT for each patient were retrieved from the report of the multidisciplinary tumor board (MTB). The study group (comprising AB, CZ, BM, JN, and SL) categorized the indications for dCRT. These were: cervical tumor location (defined as a distance between the upper esophageal sphincter and proximal tumor boundary ≤5 cm), irresectable disease (T4b tumor or irresectable lymph node metastases), unfit for surgery, and patient and/or physician preference.

### Disease recurrence

Electronic patient records were used to identify whether patients had recurrences of disease. Recurrence was categorized as either locoregional disease (i.e. involving the esophagus and/or locoregional lymph nodes) confirmed with tissue biopsies or distant recurrence (i.e. involving non-regional lymph nodes or organs) detected on (PET)/CT-scans and/or biopsies. In cases of uncertainty about the location of recurrent disease, the study team made the final decision based on radiological images.

### Treatment after recurrence

The type of treatment after recurrence was identified from electronic patient records and could involve salvage surgery, palliative systemic anti-tumor therapy, radiotherapy, or best supportive care only. Patients who were eligible for salvage surgery underwent either transthoracic esophagectomy with gastric conduit reconstruction and cervical or intrathoracic anastomosis, transhiatal esophagectomy with gastric conduit reconstruction and cervical anastomosis, or laryngopharyngectomy with jejunal interposition. Open, hybrid, and totally minimally invasive techniques were used, including robot-assisted approaches. In patients with recurrent disease in whom salvage surgery was not feasible, palliative therapy was offered, including chemotherapy, a combination of chemotherapy and immunotherapy, radiotherapy, or best supportive care.

### Study outcomes

The primary outcome was overall survival (OS). The secondary outcomes were the proportion of patients that completed the full dCRT regimen (defined as at least 100% of intended chemotherapy cycles and 90% of intended radiotherapy fractions, permitting dose delay), 30-day and 90-day mortality, the proportion of patients with recurrent disease or distant metastases after dCRT, including the location of recurrence or metastases and the recurrence-free survival, the proportion of patients that underwent esophagectomy or palliative therapy after detection of recurrent disease or metastases, and the proportion of patients undergoing endoscopic dilatation to palliate dysphagia.

### Statistical analyses

Patient and tumor characteristics were analyzed using descriptive statistics and presented as mean, median with interquartile ranges (IQR), or frequencies (%). OS was calculated from the start of dCRT until the date of death due to any cause or date of last follow-up, using the Kaplan–Meier method with the log-rank test for significance. The proportion of patients that completed the full dCRT regimen, as well as the proportion of patients with recurrent disease or distant metastases, and the proportion of patients that underwent palliative therapy or esophagectomy after detection of recurrent disease, were calculated relative to all patients treated with dCRT. Median time to recurrence was calculated from the date of the last cycle of dCRT until disease recurrence. The recurrence-free survival was calculated from the date of the last cycle of dCRT using competing risk analysis to correct for the presence of competing events (i.e. death). Univariable and multivariable Cox regression analyses were used to determine which variables predict survival, including sex, age, tumor type, differentiation grade, clinical T-category, clinical N-category, WHO performance status, and number of comorbidities (i.e. diabetes, myocardial infarction, chronic obstructive pulmonary disease, and cerebrovascular accident). The results of the cox regression analyses were expressed using hazard ratios (HR) with their corresponding 95% confidence intervals. A *P*-value <0.05 was considered statistically significant (two-sided). Statistical analyses were performed using SPSS software, version 28.0 (SPSS, IBM, New York, NY, USA) and R version 4.0.4 (www.r-project.org).

## RESULTS

**Fig. 1:**
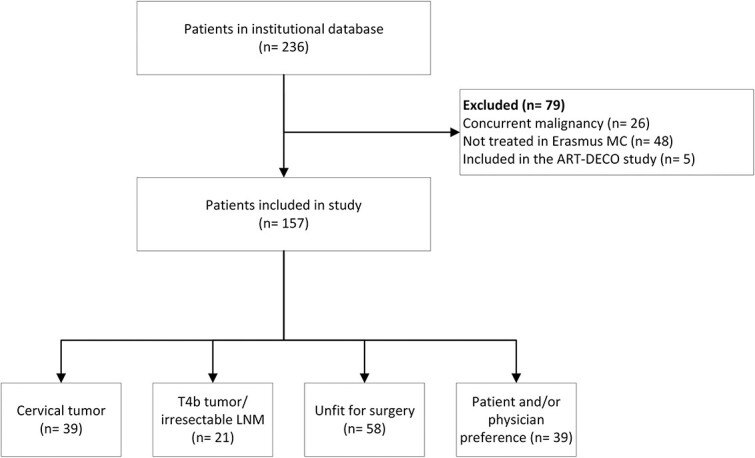
Study flowchart of patients who were treated with dCRT for various indications**.** LNM, lymph node metastases.

In total, 236 patients were identified from the institutional database. After exclusion of 79 patients (*n* = 26 concurrent cancer diagnoses, *n* = 48 treatment with dCRT in another hospital precluding access to post-therapy outcomes, and *n* = 5 treated with 61.6 Gy in the ART-DECO study),^11^ 157 patients were eligible for analyses of the primary and secondary outcomes (Fig. 1). The indications for dCRT were cervical location of the tumor (24.8%), irresectable disease (T4b tumor or irresectable lymph node metastases) (13.4%), unfit for surgery (36.9%), and preference of the patient and/or physician (24.8%). Patient and tumor characteristics are listed in Table 1.

**Table 1:** Baseline characteristics

Characteristics	All patients (*n* = 157, %)
Sex Male	93 (59.2)
Age (median [IQR])	69 (63–75)
Tumor type Adenocarcinoma Squamous cell carcinoma	43 (27.4) 114 (72.6)
Tumor location Cervical esophagus^*^ Proximal esophagus Middle esophagus Distal esophagus/EGJ	49 (31.2) 21 (13.4) 36 (22.9) 61 (38.9)
Differentiation grade Well differentiated (G1) Moderately differentiated (G2) Poorly differentiated (G3) Missing	12 (7.6) 76 (48.4) 23 (14.6) 46 (29.3)
Clinical T-category cT1/T1b cT2 cT3 cT4/T4a cT4b cTx	4 (2.5) 26 (16.6) 95 (60.5) 4 (2.5) 19 (12.1) 9 (5.7)
Clinical N-category cN0 cN1 cN2 cN3 cNx	42 (26.8) 70 (44.6) 32 (20.4) 9 (5.7) 4 (2.5)
WHO performance status 0–1 ≥2 Missing	141 (89.8) 13 (8.3) 3 (1.9)
Number of comorbidities 0–1 ≥2	143 (91.1) 14 (8.9)

IQR, interquartile range; EGJ, esophagogastric junction.

^*^ Cervical tumor location was defined as a distance between the upper esophageal sphincter and proximal tumor boundary ≤5 cm.

### Survival

**Fig. 2:**
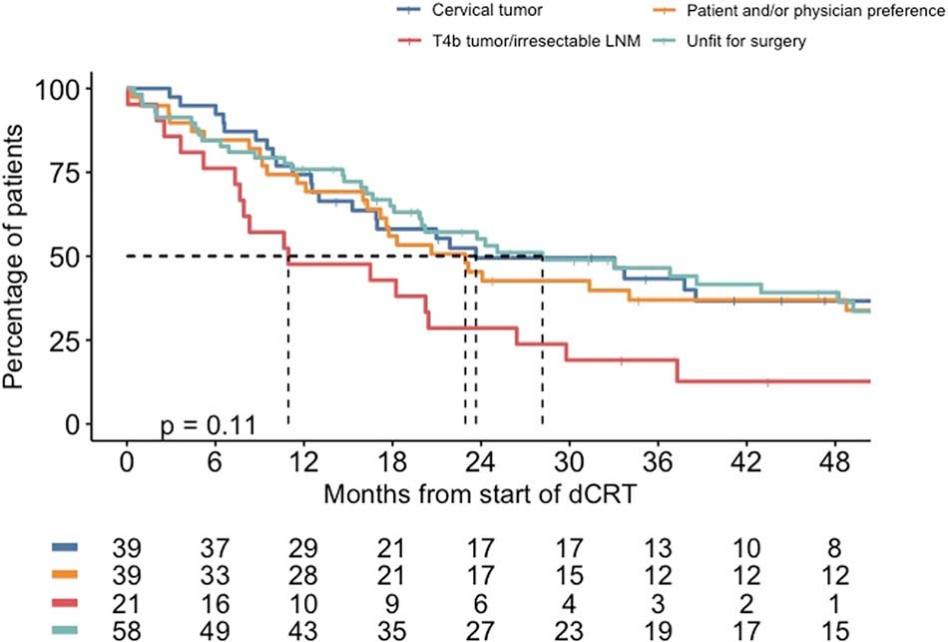
Overall survival after definitive chemoradiotherapy for subgroups**.** LNM, lymph node metastases.

The median follow-up was 20.2 months (IQR 10.0–43.9). Median OS of all 157 patients was 22.9 months (95% CI 18.0–27.9), with corresponding 3- and 5-year survival rates of 39.4% and 31.4%, respectively. The median OS according to subgroups was 23.7 months (95% CI 6.5–40.8) for those with a cervical tumor location, 10.9 months (95% CI 0.0–23.2) for patients with irresectable disease, 28.2 months (95% CI 12.3–44.0) for patients unfit for surgery, and 22.9 months (95% CI 15.4–30.5) for those with patient and/or physician preference for dCRT (*P* = 0.11) (Fig. 2 and Table 2).

**Table 2:** Survival after definitive chemoradiotherapy for subgroups

**Predefined indications**	**Median follow-up in months (IQR)**	**3-year OS rate (%)**	**5-year OS rate (%)**
Cervical tumor T4b tumor/irresectable LNM Unfit for surgery Patient and/or physician preference	21.1 (11.2–44.4) 10.9 (6.3–28.1) 21.5 (11.7–48.1) 20.6 (9.5–54.2)	43.3 19.1 46.5 37.0	36.7 12.7 33.6 33.9

IQR, interquartile range; OS, overall survival; LNM, lymph node metastases.

The median OS for patients that did not complete the full dCRT regimen was 16 months (95% CI 9.2–22.8). The median OS for esophageal squamous cell carcinoma and esophageal adenocarcinoma was 24.1 months (95% CI 14.07–34.1) and 17.6 months (95% CI 12.52–22.64), respectively (*P* = 0.1). Median OS was 20.2 months (95% CI 14.7–25.8) in patients in whom the tumor extension or location precluded surgery (i.e. cervical location or irresectable disease), compared to 24.1 months (95% CI 13.2–35.0) for patients unfit for surgery or own/physicians preference (*P* = 0.42).

Univariable analysis revealed no significant association between patient and tumor characteristics and OS (Table 3). Therefore, no multivariable analysis was performed.

**Table 3:** Univariable cox regression analyses for survival after definitive chemoradiotherapy

	Hazard ratio (95% CI)	*P*-value
Gender Male Female	1 0.722 (0.493–1.057)	0.090
Age (yrs.) <65 >65	1 0.737 (0.502–1.082)	0.119
Tumor type Squamous cell carcinoma Adenocarcinoma	1 1.387 (0.931–2.067)	0.116
Differentiation grade Good Moderate Poor Missing	1 2.053 (0.886–4.758) 1.878 (0.743–4.475) 1.428 (0.593–3.442)	0.093 0.183 0.427
Clinical T-category cT1/T1b cT2 cT3 cT4/T4a cT4b cTx	1 0.813 (0.238–2.780) 1.114 (0.349–3.553) 1.694 (0.341–8.415) 1.862 (0.541–6.404) 1.406 (0.363–5.445)	0.742 0.855 0.519 0.324 0.622
Clinical N-category cN0 cN1 cN2 cN3 cNx	1 0.857 (0.549–1.339) 0.910 (0.533–1.553) 0.880 (0.388–1.996) 1.540 (0.469–5.055)	0.499 0.728 0.760 0.476
WHO performance status 0 1 ≥2 Missing	1 1.146 (0.671–1.956) 0.937 (0.400–2.195) 1.439 (0.406–5.107)	0.618 0.881 0.573
Number of comorbidities 0 1 ≥2	1 0.905 (0.605–1.354) 0.682 (0.340–1.370)	0.627 0.282

CI, confidence interval; yrs., years.

### Therapy completion and mortality

Of all 157 patients, 116 (73.9%) completed the full dCRT regimen (all chemotherapy cycles plus at least 90% of radiotherapy fractions), and 142 (90.4%) completed all 28 radiotherapy fractions, regardless of the number of completed chemotherapy cycles. Failure to complete the full dCRT regimen was caused by deteriorating conditions or complications of dCRT. The 30-day mortality from the start of dCRT was 2.5%, and this increased to 8.3% at 90 days. Death within 30 days was related to treatment in all patients, while treatment-related death within 90 days was seen in 41% of patients. Treatment-related deaths included aorto-esophageal fistulas and radiation pneumonitis.

### Disease recurrence

**Fig. 3:**
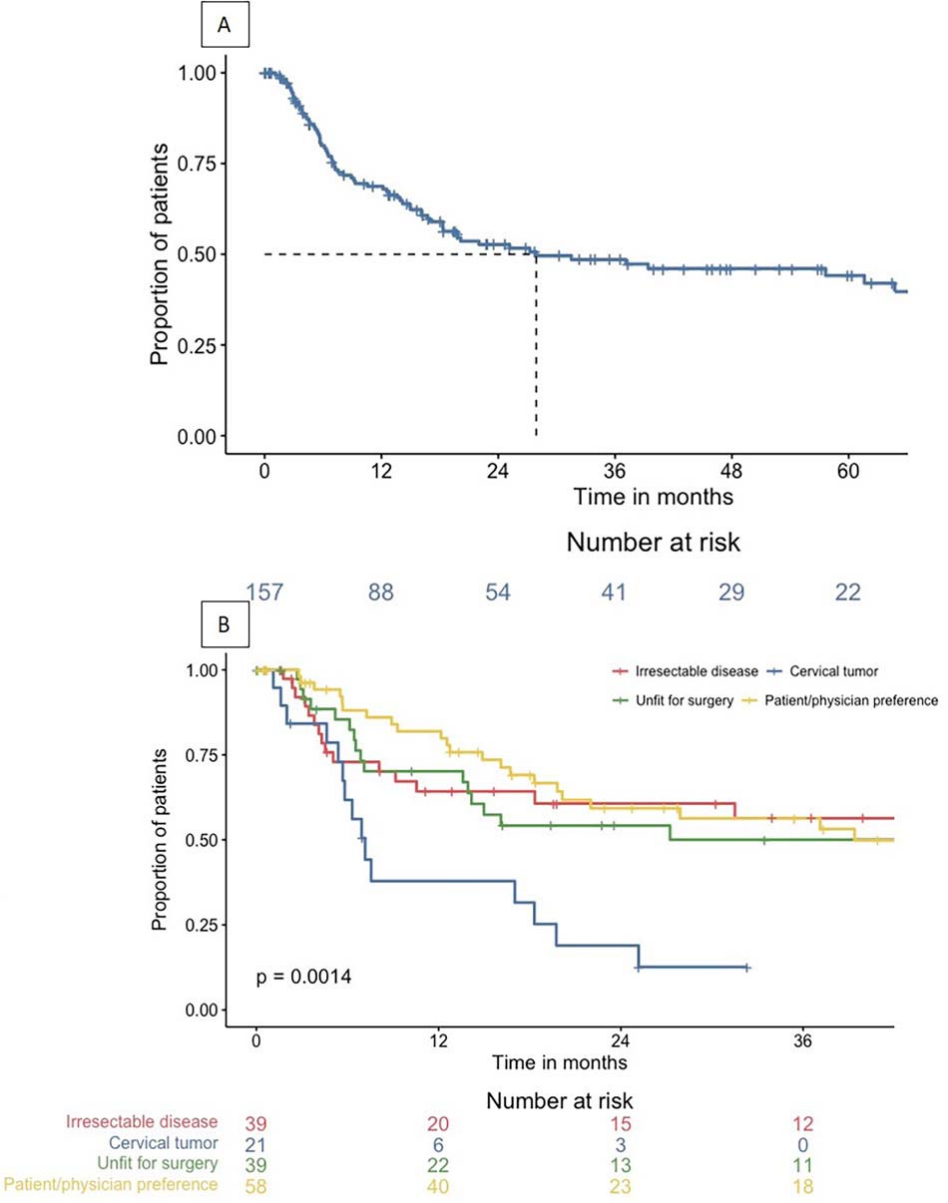
Recurrence-free survival for all patients (A) and according to indication (B).

**Fig. 4:**
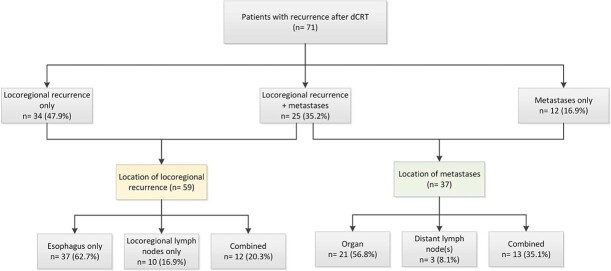
Recurrence patterns after definitive chemoradiotherapy. dCRT, definitive chemoradiotherapy.

Out of the 157 patients, 71 (45.2%) had disease recurrence, which occurred at a median of 7.2 months (IQR 4.3–16.7) after completion of dCRT. The recurrence free survival was 69% at 1 year and 53% and 44% at 2 and 5 years, respectively (Fig. 3A). Among these patients, 34 (47.9%) had locoregional recurrence only, 12 (16.9%) had metastases only, and 25 (35.2%) had both locoregional recurrence and metastases (Fig. 4). The distribution of disease recurrence according to dCRT indications is shown in Table 4. The recurrence-free survival for subgroups is presented in Fig. 3B. The distribution of disease recurrence for patients with esophageal adenocarcinoma (*n* = 22) was locoregional only in 8 patients (36.4%), metastases only in 3 patients (13.6%), and both locoregional and metastases in 11 patients (50%). For esophageal squamous cell carcinoma (*n* = 49), 26 patients had locoregional recurrence only (53.1%), 9 (18.4%) had only distant metastases, and 14 patients (28.6%) had both locoregional recurrence and metastases. For patients who did not complete the full dCRT regimen, the RFS was 76% at 1 year and 44% at 3 and 5 years.

**Table 4:** Recurrence patterns according to predefined indications for definitive chemoradiotherapy

	**Cervical tumor location, *n* = 17 (%)**	**Irresectable disease, *n* = 15 (%)**	**Unfit for surgery, *n* = 23 (%)**	**Patient/physician preference, *n* = 16 (%)**
**Location of disease recurrence** Locoregional recurrence Distant recurrence Combined locoregional and distant	8 (47.1) 2 (11.8) 7 (41.2)	5 (33.3) 2 (13.3) 8 (53.3)	12 (52.2) 4 (17.4) 7 (30.4)	9 (56.3) 4 (25.0) 3 (18.8)

Of the 71 patients with recurrent disease, 28 patients (39.4%) received therapy, and 43 patients (60.6%) received only the best supportive care.

In total, 34 patients had locoregional recurrent disease, and 7 of these patients (20.6%) underwent explorative surgery, and 4 of these 7 (57.1%) underwent tumor resection. One of these four patients underwent a laryngopharyngectomy with jejunal interposition. The remaining three patients had an irresectable tumor or peritoneal metastases at the time of surgical exploration, resulting in 4 of 34 patients (11.8%) with locoregional recurrence following dCRT undergoing treatment with curative intent (i.e. surgical resection).

Of the remaining 21 patients who underwent therapy after dCRT for recurrent disease, 10 patients (47.6%) were treated with palliative chemotherapy, 10 patients (47.6%) with palliative radiotherapy, and 1 patient (4.8%) was treated with combined palliative chemotherapy and immunotherapy. Thirteen (30.2%) of forty-three patients who had best supportive care received an esophageal stent.

## DISCUSSION

This is the first study investigating the outcomes of patients treated with dCRT according to predefined indications. This retrospective cohort study showed a 5-year OS of 31% after treatment with dCRT. Looking at patient subgroups according to dCRT indications, patients with irresectable disease had the worst survival. Nearly half of the patients developed recurrent or metastatic disease at median 7.2 months after treatment completion. The entire dCRT regimen was successfully completed by 74% of patients, but with a considerable 90-day mortality rate of 8.3%.

In current literature, OS is consistently reported to combine all indications for dCRT, resulting in a heterogeneous population. In our study, we investigated outcomes for patient subgroups according to the indication for dCRT as defined by the MTB, which makes this a unique study. By focusing on these patient subgroups, we aimed to provide more insight into the efficacy and outcomes of dCRT from a clinical perspective. If patients lack alternative options due to tumor location or irresectability, dCRT stands as the only possible curative-intended therapy. However, patients who opt for dCRT as a non-surgical treatment demonstrate a 15% lower 5-year survival rate compared to those who choose nCRT followed by esophagectomy, notwithstanding variations in patient demographics.^1^ Consequently, the combination of nCRT and surgery should be the preferred treatment strategy for this subgroup of patients, with thorough consideration of its advantages and disadvantages routinely addressed by the attending physician during patient consultations. Furthermore, when considering dCRT as an alternative treatment strategy for patients unfit for surgery, the significant 90-day mortality, high rate of premature treatment discontinuation, and relatively low chance of cure should lead to careful consideration of alternative treatment options for these fragile patients, such as palliative radiation.

The survival rates reported in our study align with findings from other studies,^7^^,^^11^ although some older studies have reported lower survival rates.^8^^,^^12^^,^^13^ The different chemotherapy regimens that were used in those studies and the associated toxicity, as well as the post-recurrence therapeutic options, may have contributed to these discrepancies.

Additionally, heterogeneity in indications and follow-up strategies across studies needs to be considered when interpreting outcomes. While our univariable analysis did not identify any significant factors influencing OS, other studies have reported T-category, N-category, tumor location, tumor stage, tumor histology, and WHO performance status to be associated with survival.^9^^,^^14–16^ It is worth noting that these studies primarily focused on patients with either cervical-located tumors or irresectable disease, while our study also included patients who opted for dCRT instead of neoadjuvant therapy plus surgery.

Only 75% of patients successfully completed the full dCRT regimen, and there was a significant 90-day mortality rate of 8%. This rate is consistent with that reported in previous studies, emphasizing the treatment-related risks of dCRT.^17^ This accentuates the importance of careful and comprehensive patient selection for dCRT and the necessity for appropriate counseling.

Locoregional recurrence and distant metastases after dCRT remain significant challenges in the management of esophageal cancer. In our study, disease recurrence was observed in 45% of patients, with locoregional recurrence being the most common (47.9%), followed by both locoregional and distant metastases (35.2%) and distant metastases alone (16.9%). The rates of locoregional recurrence reported in our study align with findings from previous studies and are markedly higher than after nCRT and resection.^7–9^^,^^14^^,^^18^ Surgery could be the pivotal element in this nCRT treatment approach, playing a significant role in lowering the likelihood of locoregional recurrent disease compared to the non-surgical approach with dCRT. It is important to consider this factor when deciding between nCRT and dCRT, as locoregional recurrence is linked to symptoms that significantly impact the overall quality of life. These conclusions are drawn from retrospective studies; however, a direct comparison between nCRT followed by surgery and dCRT to determine which regimen better enhances local tumor control is essential. Such insights are anticipated and are likely to be answered by the ongoing NEEDS trial.^10^ The findings of our study underscore the need for enhanced local and distant control strategies during dCRT to minimize the risk of disease recurrence, and can be an argument to opt for nCRT plus resection in tumors with a high chance of locoregional recurrence, given its detrimental effect on quality of life. Moreover, the presence of distant metastases in a significant number of patients following dCRT emphasizes the need for more effective systemic therapies. The majority of patients (55.4%) with recurrent disease did not receive anti-cancer therapy. Curative therapies for recurrent disease, such as salvage esophagectomy, were only utilized in a very small proportion of patients (5.4%), highlighting the scarcity of options for patients with a recurrence of disease. It should be noted that almost all patients did not have a post-treatment follow-up with an endoscopy and/or CT scan to detect asymptomatic disease.

Keeping the high locoregional recurrence rate after dCRT in mind, the ARTDECO study investigated a dose-escalation regimen to decrease locoregional disease recurrence.^11^ Unfortunately, dose escalation up to 61.6 Gy to the primary tumor did not show higher progression-free survival in both patients with adenocarcinoma and squamous cell carcinoma.

Some strengths and weaknesses of this study need to be considered. The study had a relatively large sample size with a description of recurrence patterns and, as such, gave a valuable insight into the patient’s prognosis following dCRT. Previous studies reported outcomes for a distinct subgroup of patients treated with dCRT, which makes it challenging to compare results across studies and derive overarching conclusions.^14^^,^^19^ The unique framework of our study allows us to evaluate outcomes following dCRT in patients with predefined indications for therapy. Despite its contributions, it is important to acknowledge the inherent limitations associated with its retrospective design, including missing data. For example, no data were available on the reasons (not) to choose therapy after the detection of disease recurrence, while this may have influenced survival outcomes. Another drawback is the definition of locoregional recurrence. For this study, we defined locoregional recurrence as the presence of disease in the esophagus or regional lymph nodes. However, it is important to note that we did not take into account the location of recurrent disease in locoregional lymph nodes in relation to the radiation field, which may impact the comprehensiveness and generalizability of our findings. The absence of documented toxicity during dCRT could hinder the comprehension of its potential risks, despite not being the primary focus of the study. Furthermore, a significant subset of patients was excluded due to treatment at another hospital, complicating the presentation of results for the entire cohort. Hence, conducting this study at a national level would be imperative.

In conclusion, this study provides insight into the OS and recurrence patterns in patients with predefined indications for dCRT. Even after curatively intended therapy, the overall survival of patients with advanced esophageal cancer treated with dCRT remains poor and comes with significant post-treatment mortality. nCRT followed by surgery remains the preferred treatment for fit patients. A nationwide study can be helpful to verify the findings of this study in a larger cohort.

## Notes

Charlène J. van der Zijden and Anna Bouwman have contributed equally to this work.

Specific author contributions:Conceptualization, methodology, investigation, formal analysis, writing-first draft, writing—review & editing: Charlène J. van der Zijden, Anna Bouwman; Conceptualization, methodology, writing—review & editing: Sjoerd M. Lagarde; Writing—review & editing: all authors.
